# Online exercise for the design and simulation of PCR and PCR-RFLP experiments

**DOI:** 10.1186/1756-0500-6-513

**Published:** 2013-12-05

**Authors:** Rosario María San Millán, Ilargi Martínez-Ballesteros, Aitor Rementeria, Javier Garaizar, Joseba Bikandi

**Affiliations:** 1 Department of Immunology, Microbiology and Parasitology, Faculty of Medicine and Odontology, University of the Basque Country (UPV/EHU), Sarriena s/n, 48940, Lejona, Bizkaia, Spain; 2 Department of Immunology, Microbiology and Parasitology, Faculty of Pharmacy, University of the Basque Country (UPV/EHU), Paseo de la Universidad 7, 01006, Vitoria-Gasteiz, Spain; 3 Department of Immunology, Microbiology and Parasitology, Faculty of Science and Technology, University of the Basque Country (UPV/EHU), Sarriena s/n, 48940, Lejona, Bizkaia, Spain

**Keywords:** PCR, PCR-RFLP, Exercise, Primer, Restriction endonuclease

## Abstract

**Background:**

Polymerase Chain Reaction (PCR) and Restriction Fragment Length Polymorphism of PCR products (PCR-RFLP) are extensively used molecular biology techniques. An exercise for the design and simulation of PCR and PCR-RFLP experiments will be a useful educational tool.

**Findings:**

An online PCR and PCR-RFLP exercise has been create that requires users to find the target genes, compare them, design primers, search for restriction endonucleases, and finally to simulate the experiment. Each user of the service is randomly assigned a gene from *Escherichia coli*; to complete the exercise, users must design an experiment capable of distinguishing among *E. coli* strains. By applying the experimental procedure to all completely sequenced *E. coli*, a basic understanding of strain comparison and clustering can also be acquired. Comparison of results obtained in different experiments is also very instructive.

**Conclusions:**

The exercise is freely available at http://insilico.ehu.es/edu.

## Findings

In 1993, Kary B. Mullis was awarded the Nobel Prize in Chemistry “*for his invention of the polymerase chain reaction (PCR) method”*, a technique he started to work on only a decade before, in 1983 (http://www.nobelprize.org). PCR revolutionized molecular biology, and was promptly combined with Restriction Fragment Length Polymorphism technique to yield PCR-RFLP [1]. Both, PCR and PCR-RFLP are widely used molecular biology techniques.

The purpose of this work was to develop an online learning resource for PCR and PCR-RFLP techniques. Some programs for PCR simulation are available online [2,3], but they have been created primarily for research. At any rate, their features do not include PCR-RFLP simulation. Our group also created a PCR simulation program available online since 2003 [4], which includes in its actual version (but not in the original one) a PCR-RFLP simulation program and programs to simulate other molecular biology techniques against completely sequenced prokaryotes. The service described in this work facilitates “learning by doing” through simulation of all the steps required to complete PCR and PCR-RFLP experiments, and may be considered introductory to the molecular biology simulation programs available on our website. To our knowledge, no integrated service similar to the one described in this work is available online or as a downloadable program.

Usage of the online exercise does not require registration, and several users may access the site simultaneously (for example, a class of students in a computer room). All the exercises may be completed without leaving the server, and the exercise is available in English, Spanish, and Basque. A video and a presentation explain how to complete the exercises and outline the basics of the simulated techniques.

**Figure 1:**
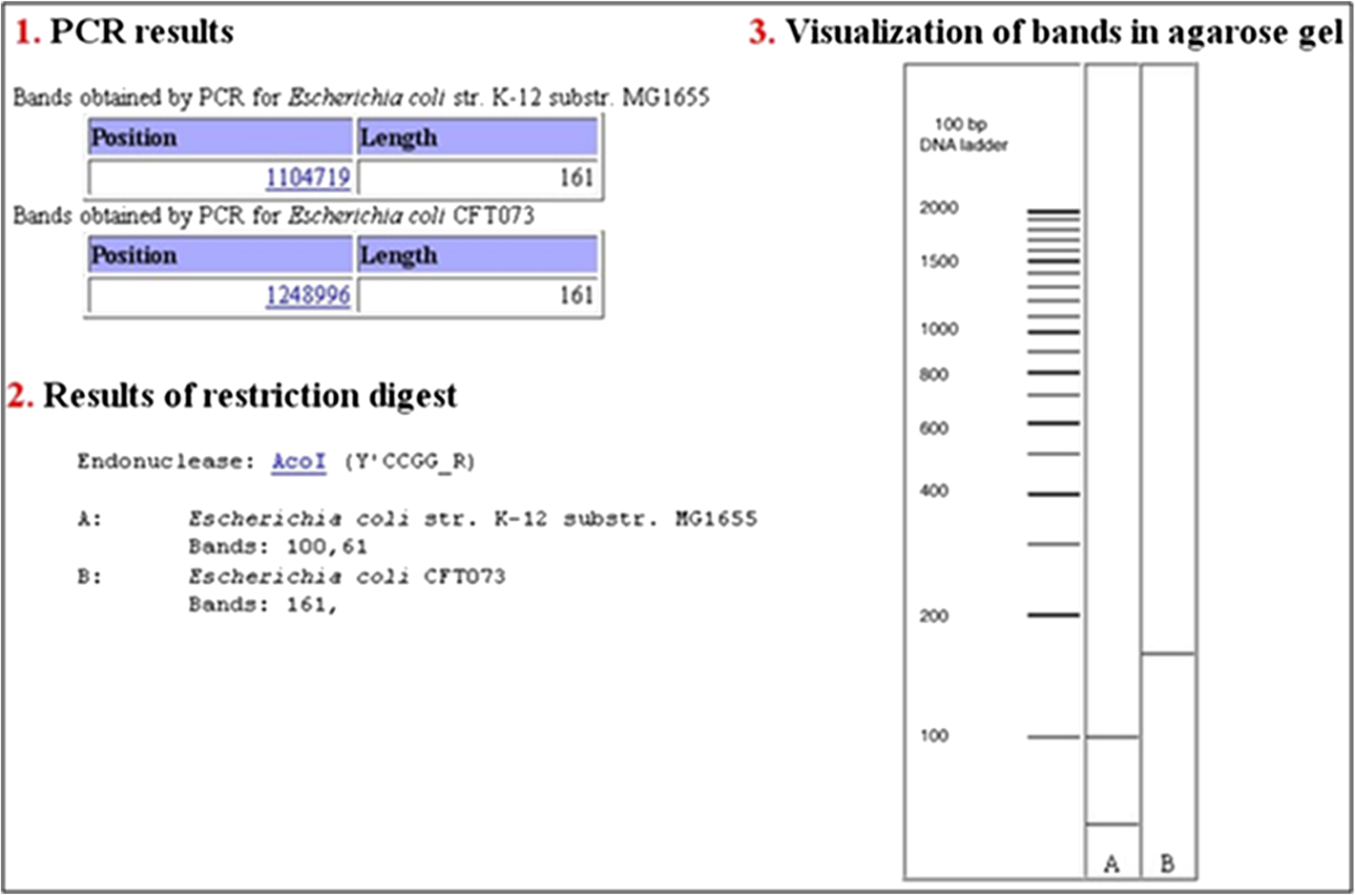
**Basic output of the exercise.** During the exercise, PCR amplification is carried out for both problem genomes with primers selected by the users (GGGCGTGATCCATTTTTATG and CTATTTGCGCGTTTTTGACA) for problem gene (*ymd*A) (1). In the PCR-RFLP exercise, after selection of the restriction endonuclease, different cleavage patterns are expected for amplicons (2), and the bands yielded will be shown in a simulated gel (3).

The service will generate for each user/session a PCR (short exercise) or PCR-RFLP (long exercise) problem: one out of 750 pre-selected genes present in two strains of the model organism *E. coli* is assigned to the user. To solve the PCR exercise, users must retrieve the selected gene sequences, align and compare their DNA and protein sequences, design primers, select the reagents for the PCR reaction, and simulate it. The PCR exercise is solved when bands are observed for both genomes in the virtual agarose gel. If the long exercise is chosen, sequences of the two amplicons obtained in the PCR experiment must be searched for restriction endonucleases that show different cleavage patterns. The selected restriction endonuclease is used for simulation of the PCR-RFLP exercise. The exercise is solved when different bands patterns are obtained for the two genomes (Figure 1). At the end of the exercise, all the information generated during the experiment can be retrieved, including the sequences of the amplicons.

**Figure 2:**
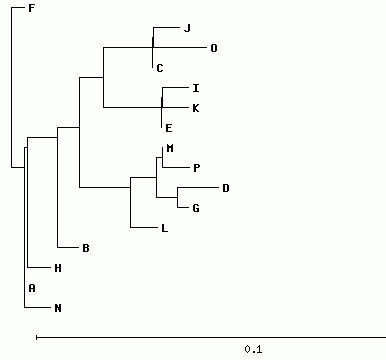
**Clustering of PCR amplicons.** Sequences from all amplicons are compared and clustered. To allow a fast response to several simultaneous users (as for example to students in a computers room) the amplicons with identical sequences are grouped and identified with a letter. In this example, amplicons obtained with primers GGGCGTGATCCATTTTTATG and CTATTTGCGCGTTTTTGACA from all 57 sequenced *E. coli* genomes are grouped into 16 groups with identical sequences (A to P).

Additionally, the program allows the simulation of PCR and PCR-RFLP techniques for all complete *E. coli* genomes available through the International Nucleotide Sequence Database (http://www.insdc.org/), as well as the comparison of the results. For PCR, the amplicons obtained for *E. coli* genomes may be aligned, compared, and clustered, and a dendrogram is generated (Figure 2). When two or more simulations are performed (for example, by several students) the results obtained in different experiments may be compared. As PCR-RFLP results and dendrograms obtained for each gene/experiment differ considerably, divergent results may be used to emphasize the importance of proper gene selection to allow clustering patterns obtained in the dendrograms to be associated with phylogenetic relationships among bacterial strains, as might be necessary, for example, during an outbreak.

The exercise’s educational benefit stems from the fact that one needs to understand the crucial steps and procedures carried out during the exercise in order to complete it, and relies upon the availability of sequences on the internet, as well as programs for alignment, primer design, and comparison of endonuclease restriction patterns or clustering. Multiple-choice questions were generated as a companion to the service.

### Implementation

The website was developed with open-source software (PHP running on an Apache server using the Linux operating system). Sequences from prokaryotic genomes and other related files were obtained from NCBI [5], and data related to restriction endonucleases from REBASE [6].

The open-source programs ClustalW [7] and Primer3 [8] were integrated in the service for alignment and primer design respectively. For comparison of restriction endonuclease patterns, and computing of distances and clustering of amplicons, specialized scripts were developed. To retrieve sequences and other data from amplicons, the service was integrated with the actualized version of the web service developed by Bikandi et al. [4]. To allow fast responses to several simultaneous users, most data used by the service were pre-computed.

## Availability and requirements

The PCR exercise is available over the Internet at http://insilico.ehu.es/edu. Its correct behavior was verified with all major browsers.

The script to search for commercially available restriction endonucleases capable of generating different restriction patterns for the amplicons obtained in the experiment was integrated with a previous script developed for the restriction of single sequences [4] and a multipurpose independent downloadable PHP program was generated (http://insilico.ehu.es/restriction/main/).

## Conclusions

An online exercise for the design and simulation of PCR and PCR-RFLP experiments was created. At the end of the exercise, the designed procedures may be applied to all sequenced *E. coli* genomes. The exercise may be completed individually or simultaneously by students present in a computer room. The latter option also allows the comparison of results obtained in different experiments, so the importance of gene selection prior to inference of phylogenetic relationships among strains may be demonstrated.

## Abbreviations

PCR: Polymerase chain reaction; PCR-RFLP: Restriction Fragment Length Polymorphism of PCR products.

## Competing interests

The authors declare that they have no competing interests.

## Authors’ contributions

Corresponding author JB conceived the project and supervised all its aspects. All authors used old versions of the service in the classroom and contributed to development of the actual service by reporting errors and suggesting modifications and new features. In specific meetings, the final design of the service and the generation of the companion video, presentation and multiple response questions were decided collectively. All authors read and approved the final manuscript.

## Authors’ information

All authors are lecturers in Immunology, Microbiology or Parasitology in three different Faculties of the same University and collaborate in research projects involving molecular biology techniques.

## References

[B1] Saiki RK Scharf S Faloona F Mullis KB Horn GT Erlich HA Arnheim N Enzymatic amplification of beta-globin genomic sequences and restriction site analysis for diagnosis of sickle cell anemia Science 1985 6 1350 1354 10.1126/science.2999980 2999980

[B2] Arányi T Váradi A Simon I Tusnády GE The BiSearch web server BMC Bioinforma 2006 6 431 10.1186/1471-2105-7-431 PMC1609187 17022803

[B3] Qu W Zhou Y Zhang Y Lu Y Wang X Zhao D Yang Y Zhang C MFEprimer-2.0: a fast thermodynamics-based program for checking PCR primer specificity Nucl Acids Res 2012 6 W205 W208 10.1093/nar/gks552 22689644 PMC3394324

[B4] Bikandi J San Millán R Rementeria A Garaizar J In silico analysis of complete bacterial genomes: PCR,AFLP-PCR, and endonuclease restriction Bioinformatics 2004 6 798 799 14752001 10.1093/bioinformatics/btg491

[B5] Benson DA Cavanaugh M Clark K Karsch-Mizrachi I Lipman DJ Ostell J Sayers EW Genbank Nucl Acids Res 2012 6 36 42 Database issue 10.1093/nar/gks1195 PMC3531190 23193287

[B6] Roberts RJ Vincze T Posfai J Macelis D REBASE–a database for DNA restriction and modification: enzymes, genes and genomes Nucl Acids Res 2010 6 suppl 1 D234 D236 19846593 10.1093/nar/gkp874 PMC2808884

[B7] Larkin MA Blackshields G Brown NP Chenna R McGettigan PA McWilliam H Valentin F Wallace IM Wilm A Lopez R Thompson JD Gibson TJ Higgins DG Clustal W and Clustal X version 2.0 Bioinformatics 2007 6 2947 2948 10.1093/bioinformatics/btm404 17846036

[B8] Untergasser A Cutcutache I Koressaar T Ye J Faircloth BC Remm M Rozen SG Primer3-new capabilities and interfaces Nucl Acids Res 2012 6 e115 10.1093/nar/gks596 22730293 PMC3424584

